# A comparison of the DNA content and chromosome number of fifty human tumours.

**DOI:** 10.1038/bjc.1966.10

**Published:** 1966-03

**Authors:** N. B. Atkin, G. Mattinson, M. C. Baker


					
87

A COMPARISON OF THE DNA CONTENT AND CHROMiOSOME

NUMBER OF FIFTY HUMAN TUMOURS

N. B. ATKIN, GAIL MATTINSON AN-D MARION C. BAKER
From the Departmeent of Cancer Research, Jlountt Vernon Hospital,

Northwood, Middlesex

Received for publication December 17, 1965

THE preparation of tumour material for the measurement of the stain content
of individual cells is a relatively simple procedure, and the technique of Feulgen
microspectrophotometry can be applied to virtually all samples of fresh tumour
material; indeed, adequate data on deoxyribonucleic acid (DNA) content can
thereby be obtained not only from pieces of solid tumour removed surgically,
but also from smears consisting only of moderate numbers of interphase cells.
obtained for instance by scraping the surface of malignant lesions of the cervix
uteri (Atkin, 1964b). In contrast, chromosome studies demand material contain-
ing adequate numbers of dividing cells (not always present in small samples of
human tumour material) and furthermore are subject to the vagaries of present-
day techniques for chromosome spreading: metaphases may be poorly spread
or have lost chromosomes during the course of preparation.

The object of the present study is to investigate the relation between the DNA
content (of interphase cells, estimated by microspectrophotometry) and the chrom-
osome number of a fairly large group of human tumours. We have undertaken
this investigation for two reasons: to provide more confidence in the use of
Feulgen nlicrospectrophotometry of interphase cells for the estimation of the
approximate chromosome number of a tumour, and to study in more detail the
small discrepancy between DNA content and chromosome number, usually in
the direction of more DNA per chromosome than in normal cells, previously
observed in a group of 14 tumours (Richards and Atkin, 1960).

Fifty tumours, divided into two groups, have been studied. The main group
of 30 tumours comprises those which over the past 21 years have yielded the
most abundant well-spread metaphases; some of the tumours were suitable
for karyotype analysis as well as for chromosome counts, and the DNA data
will be considered in relation to the observed karyotype abnormalities. The
second group comprises tumours on which only a few exact chromosome counts,
or mainly inexact counts, have been obtained. In part, the tumours in this
group have been selected on the basis of their DNA content: since the first
group contains only a few tumours with high DNA modes, several such tumours
have been included in the second group (recent improvements in techniques for
chromosome analysis have facilitated this, although a sparsity of metaphases
may remain a difficulty).

MATERIALS AND METHODS

To avoid possible bias, DNA estimations and chromosome counts were made
independently, by N. B. A. and G. M., and by M. C. B. respectively. The DNA
estimations were made on Feulgen-stained smears prepared from fresh tumour

N. B. ATKIN, GAIL MATTINSON AND MARION C. BAKER

tissue, or ascitic fluid containing malignant cells, as previously described (Atkin
and Richards, 1956). A few of the chromosome counts were made on squash
preparations after pretreatment in 10% " Versene ", but the majority were made
on air-dried preparations after incubation of the tumour material in the presence
of " Colcemid " and in a hypotonic medium (Atkin and Baker, 1966).
Estimation of the basic DNA value

Previous findings with the present technique were confirmed: wheni the
DNA contents of a relatively small random sample of say 30 interphase tumour
cells are measured, it is nearly always found that the majority are grouped
together, although the level of the mode varies widely in different tumours. A
few cells may be expected to be synthesising DNA and to have values up to
twice the "resting " value for this reason, but clearly these usually form only a
minority; sometimes, there are prominent secondary modes consisting of cells
with two and perhaps four times the basic value, presumably a consequence of
a high incidence of polyploidization of the basic cell-line. In nearly all the tumours
in this study, 30 interphase cell measurements were regarded as sufficient to
establish a modal value (although in fact more were usually measured). 20 or
more of these cells being within ?150% of a mode. Representative histograms
of the DNA contents of random samples of interphase cells from human tumours,
obtained with the present technique, have appeared in previous publications
(Atkin and Richards, 1956; Atkin, 1960, 1962, 1964a, 1964b).

The basic DNA value (Atkin, Richards and Ross, 1959) was calculated as
follows. Individual measurements were first plotted as a frequency distribution.
The mean stain content, in arbitrary units, of 20 or more (on the average, 30.3)
interphase cells falling within ?150% of the mode was then calculated. as was
the mean stain content of 15 to 20 leucocytes or fibroblasts, which served as
controls. The basic DNA value is the ratio of these two means (tumour/control)
x 100.

From the basic DNA value, an " expected " chromosome number (assuming
the same mean DNA content per chromosome as for normal cells) was calculated.
In this calculation, allowance has been made for the apparent difference in DNA
content between normal epithelial cells and cells of mesenchymal origin: using
the present microspectrophotometric technique, uterine epithelial cells wvere
found to have about 100% more stain content than leucocytes or fibroblasts (Atkin
and Richards, 1956). In order to define this difference more closely, data from
3 specimens of non-malignant uterine cervical epithelium and 2 of endometrium
have been summated (histograms of DNA values from all except one have been
previously published: Atkin and Richards, 1956, Fig. 3-6; the other specimen
is of normal cervix from a patient aged 41 who underwent a hysterectomy following
a tubal abortion). The ratio of the mean stain content of 210 epithelial cells
to that of approximately the same number of leucocytes and fibroblasts was
1-0987 (standard deviation ?0 0378). In calculating the standard deviation,
the following procedure was adopted: the ratio of the mean stain content of
10 epithelial cells to that of 7-10 leucocytes or fibroblasts situated near to them
in the preparation was first calculated, and the final ratio with its standard
deviation was calculated from a series of such ratios; allowance was thereby
made for possible regional variation in intensity of staining. It appears therefore
that the difference between uterine epithelium on the one hand and leucocvtes

88

TUMOUR DNA AND CHROMOSOME NUMBER

and fibroblasts on the other is very close to 100%. A similar difference has beeni
found for other types of epithelium, and for epithelium in the growing as well as
the resting phase (unpublished data). Thus, the ratio of the mean stain content
of 30 epithelial cells which were within ?150% of the main mode (ignoring cells
with higher values which were probably synthesising DNA), derived from endo-
metrium showing benign hyperplasia with frequent mitoses, to that of 30 leuco-
cytes was 1-089 (standard deviation 4?0-010).

Wre have therefore applied a correction factor of -I in calculating the expected
chromosome number from the DNA value where the tumours are of epithelial
origin. This factor has also been applied for the two seminomas, although we are
uncertain whether a similar difference applies to the homologous normal cells.
It has been applied for the teratoma, since the data on this tumour appertain
to the carcinomatous elements. Thus, for epithelial tumours:

basic DNA value   46
expected chromosome number        100       X  11

For the reticulum cell sarcoma, however, the factor 1 1 was omitted, on the
assumption that the homologous normal cells have the same mean stain contenlt
as the control cells.

The ratio of the DNA content, in terms of" expected chromosome number ",
to the observed chromosome number (conveniently called the DNAl/chro9noso8ne
ratio) provides a measure of the average DNA content per chromosome.

RESULTS

A. Main Group of 30 Tumour8

The chromosome counts obtained from each tumour are plotted as histograms
in Fig. 1. A few counts fell outside the ranges shown in the histograms. Low
counts which probably represented broken metaphases were occasionally encoun-
tered. There were usually one or two metaphases with about double the modal
value, but only in Tumour No. 3 did these form a significant proportion of the
total (the high counts have been included in the histogram), and it was noted
that in this tumour there was also a correspondingly prominent secondary mode
in the DNA distribution. On the whole, the distributions of chromosome counts
are fairly symmetrical, and the mean and modal chromosome numbers usually
coincide. There is generally no excess of hypomodal over hypermodal counts
such as might be expected if there were a significant number of incomplete meta-
phases which had lost a few chromosomes during the course of preparation.
Only No. 6, 19, 20 and 27 show some asymmetry to the left. Two tumours
(No. 8 and 9) however show a slight asymmetry to the right, with more hyper-
modal than hypomodal counts. For comparison with the DNA content, we have
taken the mean chromosome number of these two tumours, the mean being in
both instances one higher than the mode. No. 14, 21, 25 and 28 have no clear
mode, and here the mean value of the counts in the modal range has been taken
as representative of the tumour.

Brief clinical and pathological details of the cases are given in Table I, together
with the modal (or mean) chromosome number, calculated to the nearest chromo-
some (or for tumours with less than 52 chromosomes, to the nearest half chromo-

19

N. B. ATKIN, GAIL MATTINSON AND MARION C. BAKER

0

0  0

t r M W0 n0

$-. o   N n =Nt  oct

~00

0
0 -

0~~~~~~~~~~~1

C)

0      to LO x

0

0~~~~~~~~~~~~1

C)

-  C)O0   COOCO O0 .00 _cOO _ _

~ C

0 .40      - , -4

'0l

,- CO C* CO
10 10 101)

to u

o 11010  10 co

o0d0     - >i
-4 - -    -

I I     1111          0 I I I ;  P 4 t-  I I I I

CO

00P-

...   't      .1 C) -a

0 c..

.@
Ow;

0000f      .6e
OQOHQ 0

10-0~ 101-:
r.~410   10 C

C I O e10= es   r

P-  I   I_  _4  _ 41

90

+4- 0
o

oo

0.4 .2

*j -4 |

C) =

Od

*) II,,

;.,  ce

rV4

? .2
cc

0 11

C)

.oq

0

C )

*2 ^
c3

o

*C  1

eb

"-.Z

o)
o

-.b

C.)

o. z

o

0 ~ ~ ~  0

CO~~~O

~~~~~ X
;3       Ps ?3$

000 C00)     0 r  o

?ooo s oooQX soo~.4 0 -  C

144 c  10 0   w   tokm   w cfl

P- 1

TUMOUR DNA AND CHROMOSOME NUMBER

O 0    (Mm   O    0 o c   eel obc
co co_coC    - o        1ooO

__     oo   0  oN  00  _

0o         - o  00   -  " o

*~~~~

000 k m         10t  Q   4   c r  e  C  0 O

0

o

|4C~   t- CC  CC10o Q

0
0

00
0
0

0~~~~~~~~~~

1~~~~4 0~4~

4a  0 ~~~~~

0 T >~~~~~~

00   .4   0 0 4fp

111  1  11co  1cocom  1  11   11 C

X 4 0C S   1  C 0o 0   L'-1

(m   0   -   01  CC 44  10                           c o  0

0101             01010101 c                            011C

91

0    O

eq C4 C

N. B. ATKIN, GAIL MATTINSON AND MARION C. BAKER

C-  D                     5

ca. cervix

uteri

I            .    TI-    .          n     n-   n

30         40      46  50           83      90

Chromosome number

54f -           Dreticulum

FL (cell sarcoma

C1 ID       12

ca. cervix

5-           1lh           uteri

CJ D       13

10_                       ca. colon

5                 j C  D    14

5              [1- -~1F ~ ca. cervix

Ih rn n    uteri
10~ ~ ~ -D             15

S                 r      ca.corpus

uteri

.  n. _o..    r _ S

DJC        16

testicular
teratoma
uC30    40  46  50     60

Chromosome number

uv

0

6

z

C1 ID                  10

I',  10,          ca. corpus

uteri

30          40      46   50              88

Chromosome number

Chromosome number

Fia. 1.

92

0

0
z

i

2!
i

;K
p

1!
K

TUMOUR DNA AND CHROMOSOME NUMBER

IC   1clD    2? ca. ovary

1 rUlRfhTi n n r=r  I

DI  IC             22

_             ca. cervix

uteri

*    ? 4 1 1   4 ] R

. . .. . .... . . .I .I.                                              ..

Dijr                        23

_IT I} h n             semi noma

17    1l    I n-

Di IC

24

rIiFFA  in,          ca. ovary

Cj 1D               25

-L .   i 1 J 1 L

ID

ca.stomach

( 10)

l(I mph node
r,rm 1- -l  20)

Cj ID                 26

_rFI1rJl14T~  m   ca. colon

frT    m r 1 n    I

;n        fAn         7n         on

7C

Chromosome number

21

I I
z

In

o             C| ID        27

2' ca. bronchus

I             1I

0                1C&D      28

ca.cervix
Ii FF1 n nd,m T l   uteri

o  ID               C      29

ca. rectum

5                 -

,1  ,  .  ll n.f T hT,       j

43    60      70     8       90

1D  IC          30

S .seminoma

5-n         hrh

la

vu             90            90            100            110            120

Chromosome number

FIG. 1.-Exact chromosome counts from main group of 30 tumours. C = observed chromosome

number (mode, or mean of modal range). D = expected chromosome number, estimated
from DNA measurements.

some), the basic DNA value, the expected chromosome number, and the ratio
between this value and the observed chromosome number (the DNA/chromosome
ratio), calculated as described in the previous section. The modal DNA values,
in terms of expected chromosome number, assuming the same mean DNA content
per chromosome as for normal cells, are also indicated by arrows in the histograms
(Fig. 1). It will be seen from Fig. 1 that the expected chromosome number of
most of the tumours is higher than the observed chromosome number, indicating
that the mean chromosomal DNA content is greater than normal.

B. Second Group of 20 Tumours

Distributions of exact and approximate counts, together with the other
relevant data are shown in Table II. Although there were fewer exact counts
compared with the first group, it was still clear that they tended to be grouped
together; as however a mode was not clearly definable, the mean chromosome
number, to the nearest whole number, was calculated. In calculating this mean,
metaphases on which approximate counts were made were regarded as having the
central number of their class.

to

93

0

-!

0

z
z

11

??l
I                                               11 I   I

51

1.

IC

5

51

-L

51

cza             b

-;A

N. B. ATKIN, GAIL MATTINSON AND MARION C. BAKER

0

"- I I I 1-

10

4io I I I I1-

0

to

to

100I    I  -
4-

cq 11    1

Q

I    I   I   I   I   I

I  I J

1c I I I I   CZI cD_

1 1

10
o

-q I I I        I    z  14

b; I -I1I

b Ij I1-

I II  I- rQ   jO  |  | Cl_
IIIrll   I  C]|IC

I -Il_ ~I rl _ II__
I I rII  OI CICQ|I

I-I- 1  t- I IlI
I I I    -1 1i
I I I II o 1J I-

t-   I

It-  I  I  I
tCC  I  I
t_   I  I

m    I  I a,
171  I  I  1s1
t-   I    I

oo  Iq  I I

t1  1-  1

I    I

r   I  I
XO   __-I

w       I  I
UCl    I -1I

=   _q  I  I
CD      I

0r '00 C  ' -4O  0 C* 4

CO CC 4   I* * Nt "di

I-

10

I -4

01

CO  a
~ 00

I  0 1 a

II a,Cq

I 1-
I IeI

I I *I

1  1 1
1  1 1

0

0
0

0)

o

4    0

0   4

?

4Q:

o   0

o

4a

.p

H

10
m

10
to

10

C1

U:

0

10
CO

r-
, d

Cto

_

I-
01

0-

01

0-
-4

-4
-4

-4

O  r- _

00

t- C-O I
w      I
00     I
CO     I

. Oj

a &

D'_

"D01-   CO m   10 1i

COCr-CO CO C

t0o rt.        a0          0 o
* d 1          *          10 l

94

,

I

TUMOUR DNA AND CHROMOSOME NUMBER

0 E

; .  0

0 2

u Xe

o0

tt  IIE

0

,Z S Ci?

'IQ

~~~ 0 ~~c e

?  -4

*   nt.

X   I_ t

~ 0;

?    I

q 0

0

0 4

m 0   0

Q

0 0

0     0

O1~ *~ ~

0 -

0

4-f

10a

0

0

la0

C) r-

s00

0

_e

10 CO 0101 _ 100 EU- 01-- e>

O _O 00 CO- 00 4 0- 01 0

-  CO  o1 10 o 4 _   t- O   0 0D cn 1t- cz

CO C -o  o  _O CO  +  o  Ce1 CO   CO a-

0000w=CO 0

1,l00  CZ 0 CO 1

t   to  E   C   c O  c O  0

- - - 1 - 01   C

0   ~ ~ ~ ~ ~ ~ ~ ~ ~ ~ ~   ~~~0   0

S,   S                      -  -

C   ? v

>  ;  0 0  0 00 0  0 >  0 0

0Z ( 0 0 0 0 0 0 0  0 0  &  0 0  1 o  o   o0

Q O O O O O O O O O O Q C   i d   eo

bo W- =    CO  O c in C0  CO 0 = _
?-! = 0 L- to Id o 0 L- 10 10 e= Id4 u

C   + CO CO C     d 0

10         t - r- 1*  10

0 fs

r CO CO CO CO CO CO CO CO CO X

95

N. B. ATKIN, GAIL MATTINSON AND MARION C. BAKER

The relation between DNA content and chromosome number

Considering first the main group of 30 tumours on which substantial numbers
of chromosome counts were made, it is evident that except for Tumour No. 29
there is quite good agreement between basic DNA content and chromosome
number. For Tumour No. 29, however, the observed chromosome number is
approximately twice that expected from the DNA data. This discrepancy is
possibly due to the fact that in the region selected for chromosome counts most
of the cells had doubled their chromosome complement, whereas in the region on
which DNA measurements were made there was only a moderate degree of
polyploidization of the " basic " cell-line (a varying degree of polyploidy in
different regions of some human tumours has previously been inferred from data
on DNA content and nuclear size (Atkin, 1962)). DNA estimations were therefore
made on the material, which had been pretreated for chromosome spreading,
from this region: although the preparations were less satisfactory from the
point of view of identification of cell-types because of the hypotonic pretreatment
and air-drying, it was clear that the majority of interphase tumour cells in this
region of the tumour had near-tetraploid DNA contents.

Excluding Tumour No. 29, and the metastasis of Tumour No. 25 which yielded
only a few chromosome counts, the mean DNA/chromosome ratio of the remaining
29 tumours is 1-040 (standard deviation ?0-063). If we consider only the 29
untreated primary carcinomas and teratoma (excluding the two seminomas
because of uncertainty regarding the DNA content of the homologous normal
cells, i.e. whether, like epithelial cells, they have a 10% higher content than
leucocytes), we find a mean DNA/chromosome ratio of 1-026 (standard deviation
?0 042). On the other hand 4 out of the 5 tumours (No. 1, 2, 18, 21 and 27)
which were either metastases or recurrences following treatment had DNA/
chromosome ratios greater than 1-1. The DNA/chromosome ratios are shown
graphically in Fig. 2.

The data on the 20 tumours in the second group (Table II and Fig. 2) also
show a tendency for the tumours to have more DNA per chromosome than normal.
There is however slightly greater variation in the DNA/chromosome ratios than
in the first group, probably because fewer chromosome counts were made. The
mean DNA/chromosome ratio of the group is 1-049 (standard deviation -40*10).

A comparison of the DNA/chromosome ratios and the karyotypes of 17 tumours

Karyotype analyses have been performed on 17 of the tumours in the present
study, and it is of interest to compare the karyotypes with the DNA/chromosome
ratios. Karyotypes of 4 of the tumours have been illustrated in a separate
publication (Atkin and Baker, 1966): No. 11 (Fig. 4); No. 17 (Fig. 2); No.
18 (Fig. 1); and No. 19 (Fig. 3). Brief details of the karyotypes of the following
tumours are also given in Table 6 of the same publication, the numbers in brackets
being those given to the tumours in this table: No. 1 (6), 2 (7), 4 (9), 5 (8), 6 (10),
8 (11), 9 (12), 10 (13), 13 (14), 15 (16), 16 (15) and 23 (17). Tumour No. 6 was
exceptional in that most of the tumour-cell metaphases had apparently normal
karyotypes. Although the other tumours showed varying degrees of aneuploidy,
all the chromosome groups were usually represented, and no very great departure
from normal with respect to the mean DNA content per chromosome was therefore
to be expected. However, as a possible indication of a change in the DNA per

96

TUMOUR DNA AND CHROMOSOME NUMBER

97

chromosome, we have taken a representative karyotype from each of the 17
tumours on which analyses have been performed (all except No. 23 were from
female patients) and counted the numbers of " large ", " medium " and " small "
chromosomes in each karyotype (the " medium " group comprises the C group

5

0

E

0

z

5

090         1-00        1-0

DNA/chromosome ratio

1-20

FIG. 2. DNA/chromosome ratios of (A) 29 tumours from the main group (excluding No. 29

and the lymph node metastasis of No. 25) and (B) second group of 20 tumours. The shaded
areas represent recurrent or metastatic tumours.

1-3
a)

E 12
0

A

0

E i-i
0

L -1

_*_

E,,.9

0

o,

0 10

.7

6

s6
*41

2

_9  .119

. *4
17

-     *6  167 15

12
23

.13

l   l   l   I I

18

21

I                   I                  1                    I

092   096  100   104  108   112  116

DNA /chromosome ratio

1 20   124

FIG. 3.-DNA/chromosome ratios and ratios of number of large to number of small chromo-

somes (derived from representative karyotypes as explained in the text) of 17 tumours
from the main group.

A
B

1:  I n ~ = i I InTI. ~F

N. B. ATKIN, GAIL MATTINSON AND MARION C. BAKER

chromosomes and any abnormal chromosomes within the size-range of the C
group chromosomes). The ratio of the number of large to number of small
chromosomes (ignoring the medium-size chromosomes, since these are close to
the mean size) should provide some indication of changes in the average size of
chromosomes in the karyotype. This ratio for normal female cells is:

A and B group chromosomes  -   10   0 5
D, E, F and G group chromosomes  20

As can be seen from Fig. 3, the majority of the ratios are greater than 0 5, indicat-
ing relatively more large than small chromosomes compared with normal cells,
and furthermore there appears to be a correlation between this ratio and the DNA/
chromosome ratio.

DISCUSSION

Early studies on the DNA contents of human tumour cells using microspectro-
photometric techniques (Leuchtenberger, Leuchtenberger and Davis. 1 954;
Atkin and Richards, 1956) revealed that the interphase cells were usually grouped
around a mode which was either near the normal diploid level, or else near the
tetraploid level. These modes corresponded to near-diploid and near-tetraploid
chromosome numbers respectively, provided that the cells contributing to the
mode had not yet commenced to replicate their DNA, and that the mean DNA
content per chromosome was about the same as that of normal cells. However,
at the time these studies were made, techniques for studying the chromosomes
of solid tumours had not yet been developed, and it was impossible to verify
whether in fact the DNA mode of a tumour showed any correspondence to the
chromosome number. In a later study based on a series of 14 human malignant
tumours which were especially favourable for chromosome analysis, the DNA
content and chromosome number were in more or less close agreement (RBichards
and Atkin, 1960); on the average, however, the DNA content exceeded that
expected on the basis of the chromosome number by about 14%, and it thus
appeared that there might be a tendency for tumours to have a rather larger
mean chromosome size than normal cells. Up to the time of the present study,
no more detailed investigation on the relation between DNA content and chromo-
some number has been reported. Ojima, Inui and Makino (1960) found agreement
between the DNA content and approximate chromosome number of 4 uterine
tumours, but a precise comparison was not attempted.

The present study has revealed a more or less close correlation between DNA
content and chromosome number for all except one of the fifty human tumours,
although there is frequently a small discrepancy which is usually in the direction
of more DNA per chromosome than normal cells. The variation in the DNA/
chromosome ratio that we have found for different tumours is no doubt partly
due to sampling errors. The finding that the ratio is on the average about 1'04
rather than 1 might possibly be due to an undetermined systematic error. How-
ever, the observations on the karyotypes of 17 of the tumours provide confirmatory
evidence that the majority of these tumours do in fact have on the average larger
chromosomes than normal cells.

It may be instructive to consider the conditions which appear necessary for
the demonstration of the correlation that we have found in the present study.
For such a correlation to be present, it would appear that:

98

TUMOUR DNA AND CHROMOSOME NUMBER

(1) Most of the interphase cells must have similar DNA contents, i.e. the
values are grouped around a clearly-definable mode (the microspectrophotometric
technique should of course be of sufficient accuracy, otherwise a mode even if
present might not be apparent from the frequency distribution of individual
measurements).

(2) This mode is formed by cells which have not commenced DNA synthesis
and still have the " single " amount of DNA; it is not significantly distorted by
the accidental inclusion of cells which have slightly increased DNA contents
because they have commenced DNA synthesis.

(3) There is likewise a definable chromosome number, around which most of the
chromosome counts on cells in metaphase are grouped.

(4) There is a degree of consistency within each tumour: since DNA measure-
ments and chromosome counts on the solid tumours have been made on separate
pieces of tumour, the good agreement found for nearly all the tumours suggests
that generally the interphase cells in the one specimen and the metaphases in
the other have identical or almost identical chromosome complements. The lack
of correlation found for Tumour No. 2.9 indicates that there is variation within
this tumour, but, as suggested above, this variation may be merely with regard
to the degree of polyploidy; it is also of interest that although the DNA data
and clhromosome counts obtained from the primary region of Tumour No. 25
were in mutual agreement, as were those from the metastasis, the two regions
(primary and metastasis) were significantly different from each other. Examples
from the literature of human tumours and malignant effusions having more than
one modal chromosome number or DNA value have been cited elsewhere (Atkin
and Baker, 1966). However, our results suggest that mosaicism, with cell-lines
which differ widely with respect to DNA content and chromosome number, is
uncommon, at least in primary untreated tumours.

Our resuilts indicate that the above-enumerated conditions generally obtain.
and the generally close agreement between the DNA data on interphase cells
and the chromosome counts (the exception being explainable on the basis of
regional variation within the tumour) leaves little doubt that DNA estimations
on interphase cells can provide a quite accurate estimate of the total chromosomal
content of a tumour cell-line karyotype. Observations on the karyotypes, where
available, have strenghtened the conclusion that the DNA content has indeed
changed more or less in proportion to the chromosome number; in fact, the ratios
of number of large to number of small chromosomes, summarized for 17 of the
tumours in Fig. 3, have provided a useful cross-check for the observation that the
average DNA per chromosome is close to but frequently slightly greater than that
of normal cells.

Together, the DNA data and the observations on the chromosome numbers
and karyotypes of malignant cells reported here suggest the following generaliza-
tions concerning the chromosomes of human tumours. Although apparently at
random, the karyotype changes in malignant cell-lines occur within certain more
or less w ell-defined limits. It is of interest that tumours sometimes have less than
the normal diploid content of genetic material. The tumour (No. 1) with the
lowest chromosome number (38) in the present series has a DNA content that is
about 60 / less than the normal diploid value. However, the amount of genetic
material is usually increased. Tumour No. 50 has the highest chromosome
number (125), and its DNA content is proportionately even higher. Although

99

N. B. ATKIN, GAIL MATTINSON AND MARION C. BAKER

tumours may thus vary within wide limits, it is nevertheless apparent from
DNA data on a large series of tumours that certain DNA levels are favoured.
Thus, among an unselected series of 202 squamous-cell carcinomas of the cervix,
it was found from DNA data that near-diploid and near-tetraploid (usually
hypotetraploid) tumours were about equally common, whereas near-triploid
tumours were slightly less so; on the other hand, tumours with near-diploid
DNA modes predominated in a series of 51 carcinomas of the corpus uteri, although
there was also a small near-tetraploid group (Atkin, 1964a). The DNA content
of uterine tumours may be of prognostic significance: although most patients
have been followed up for less than 5 years, the data suggest a relatively more
favourable prognosis for tumours of the cervix uteri if the DNA mode is in the
hypotetraploid region and for tumours of the corpus uteri if it is in the diploid
region (Atkin, 1964a; Atkin, as yet unpublished data).

The observation that tumours frequently have on the average slightly larger
chromosomes than normal cells may perhaps reflect certain laws with regard to
the changes that have occurred in the malignant karyotype. Thus, larger chromo-
somes may be preferentially gained or smaller chromosomes lost. Structural
changes may result in the formation of two unequal-sized chromosomes, of which
the smaller is lost. It is of interest that an abnormal chromosome is sometimes
encountered in human tumours that is larger than any of the normal set-for
instance in Tumour No. 18: see Atkin and Baker (1966), Fig. 1.

The high DNA/chromosome ratio of 4 out of the 5 treated or metastatic
tumours poses the question of whether a high ratio is a common feature of recur-
rences or metastases and if so whether a similarly high ratio is present in the
corresponding primary tumour, or whether a change has occurred with the onset
of the recurrence or metastasis.

Although there may thus be a small discrepancy, the observation that the
DNA content of human tumours generally bears a more or less close relation to
the chromosome number is in keeping with the view that some degree of balance
must be maintained in the karyotype. Observations on tumour-cell karyotypes
show that most of the normal chromosome groups are represented, and abnormal
chromosomes, although present in many tumours, rarely appear to exceed 3 or 4
in number; these abnormal chromosomes may as noted above be larger than
(or sometimes smaller than) any of the normal chromosomes, but usually they are
within the normal size-range (Atkin and Baker, 1966). It is of interest to compare
the situation in tumours with that found in a representative series of 6 placental
mammals (Atkin, Mattinson, Be,ak and Ohno, 1965): despite a more than
fourfold variation in the diploid chromosome number, the mean DNA content
per cell varied by not more than about 10%. Obviously, extensive structural
changes, without significant alterations in the total chromosomal content, have
occurred during the evolution of these species and have led to the wide differences
in chromosome number. In the transition to malignancy, however, there have
been relatively few structural changes, and although the number of chromosomes
varies widely the range and distribution of chromosome sizes in human tumour
cells correspond fairly closely to those of normal cells.

This work has been supported by a grant from the British Empire Cancer
Campaign for Research. We wish to thank the staff of Mount Vernon Hospital
for providing the tumour material; Miss M. Harpour and Miss M. Sears for

100

TUMOUR DNA AND CHROMOSOME NUMBER                   101

technical assistance; Mrs. J. Thornber for preparing the figures; and Mrs. P.
Oliver for secretarial services.

SUMMARY

1. The modal DNA content (estimated by Feulgen microspectrophotometry
of interphase cells) and the chromosome number of 49 human malignant tumours
were in fairly close mutual agreement. In a further tumour, the chromosome
number was about double that expected on the basis of DNA measurements;
this discrepancy may have resulted from variation with regard to the degree of
polyploidy within the tumour, the majority of cells in the region selected for
chromosome counts having undergone polyploidization.

2. In the 49 tumours showing reasonably good agreement, there was frequently
a small discrepancy between DNA content and chromosome number: the expected
chromosome number (based on the DNA data, assuming the same mean DNA
content per chromosome as for normal cells) exceeded the observed chromosome
number on the average by about 400.

3. Karyotype observations on 17 of the tumours supported the evidence
from DNA measurements that the average chromosome size in the tumour cell
karyotypes was usually greater than normal.

4. The findings are briefly discussed in relation to the wide and apparently
random variation in the karyotypes of different tumours.

REFERENCES

ATKIN, N. B.-(1960) Acta Un. int. Cancr., 16, 41.-(1962) Cytogenetics, 1, 113.-(1964a)

Br. J. Radiol., 37, 213.-(1964b) Acta Cytol., 8, 68.

ATKrN, N. B. AND BAKER, M. C.-(1966) J. natn. Cancer Inst. (in the press).

ATKIN, N. B., MATTINSON, G., BEVAK, W. AND OHNO, S.-(1965) Chromosoma, 17, 1.
ATKIN, N. B. AND RICHARDS, B. M.-(1956) Br. J. Cancer, 10, 769.

ATKIN, N. B., RICHARDS, B. M. AND Ross, A. J.-(1959) Br. J. Cancer, 13, 773.

LEUCHTENBERGER, C., LEUCHTENBERGER, R. AND DAVIS, R. M.-(1954) Am. J. Path.,

30, 65.

OJIMA, Y., INUI, N. AND MAKINO, S.-(1960) Gann, 51, 371.

RICHARDS, B. M. AND ATKIN, N. B.-(1960) Acta Ui?. int. Cancr., 16, 124.

				


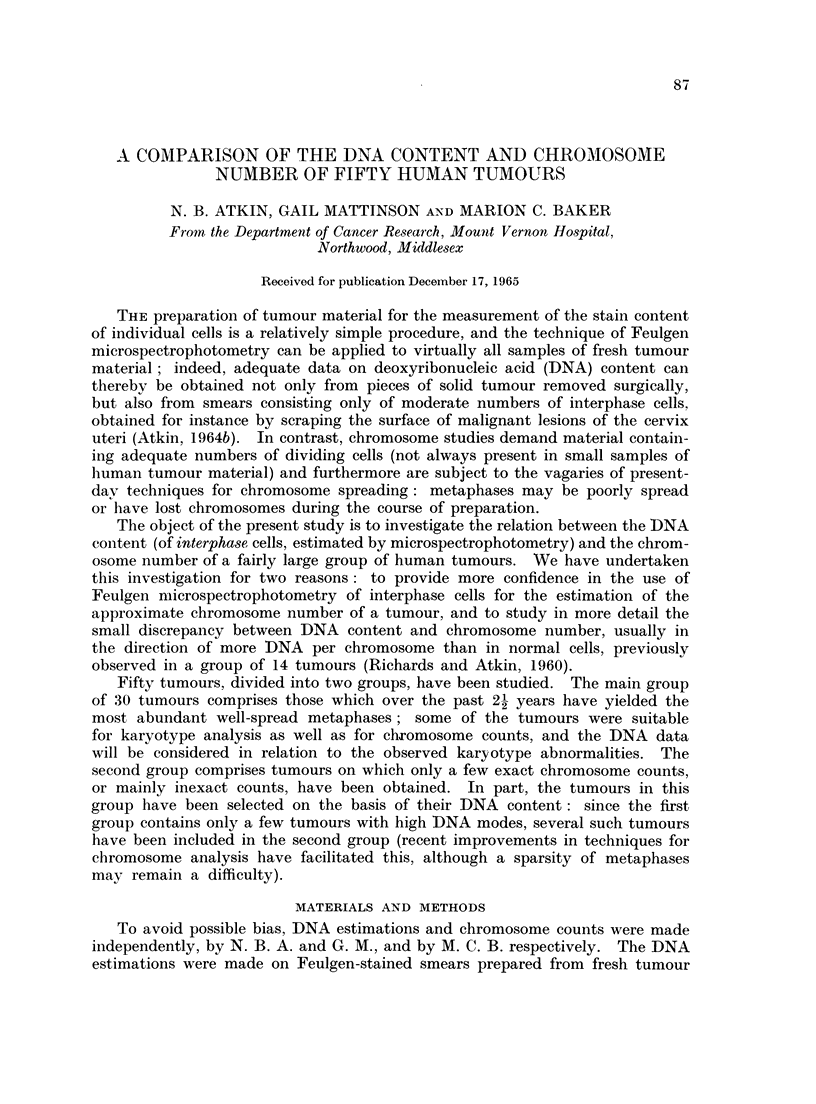

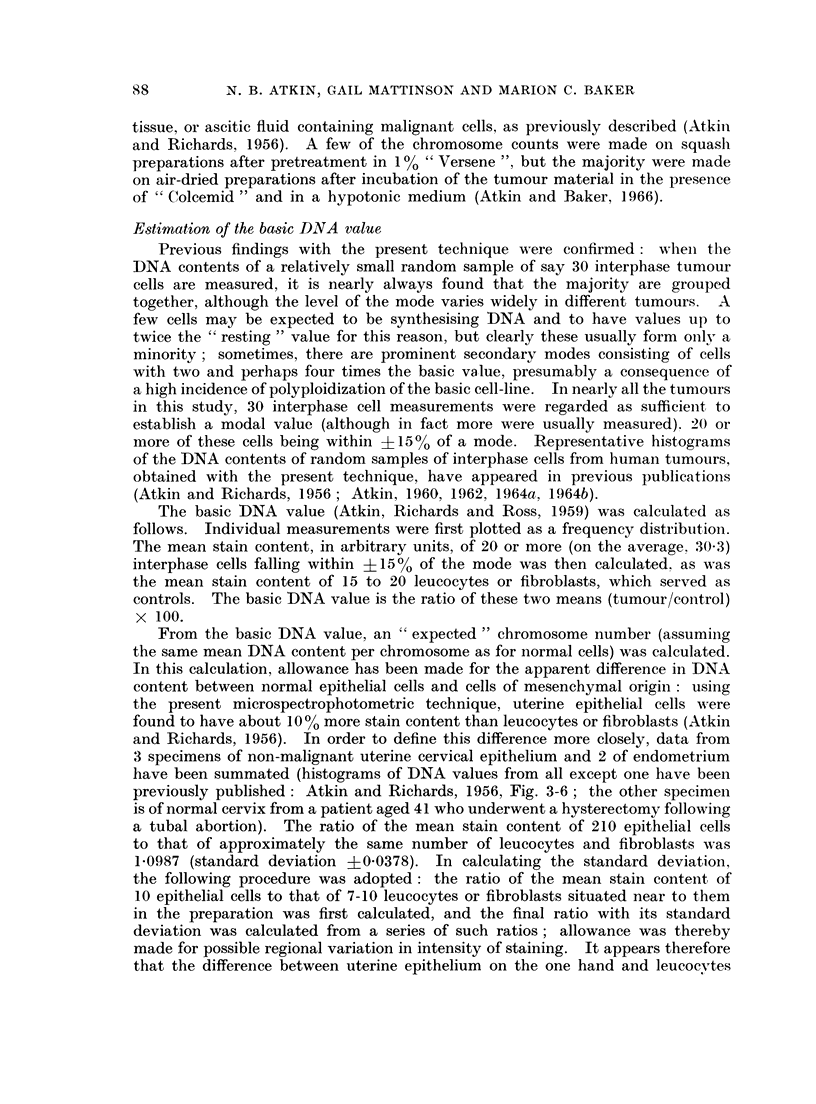

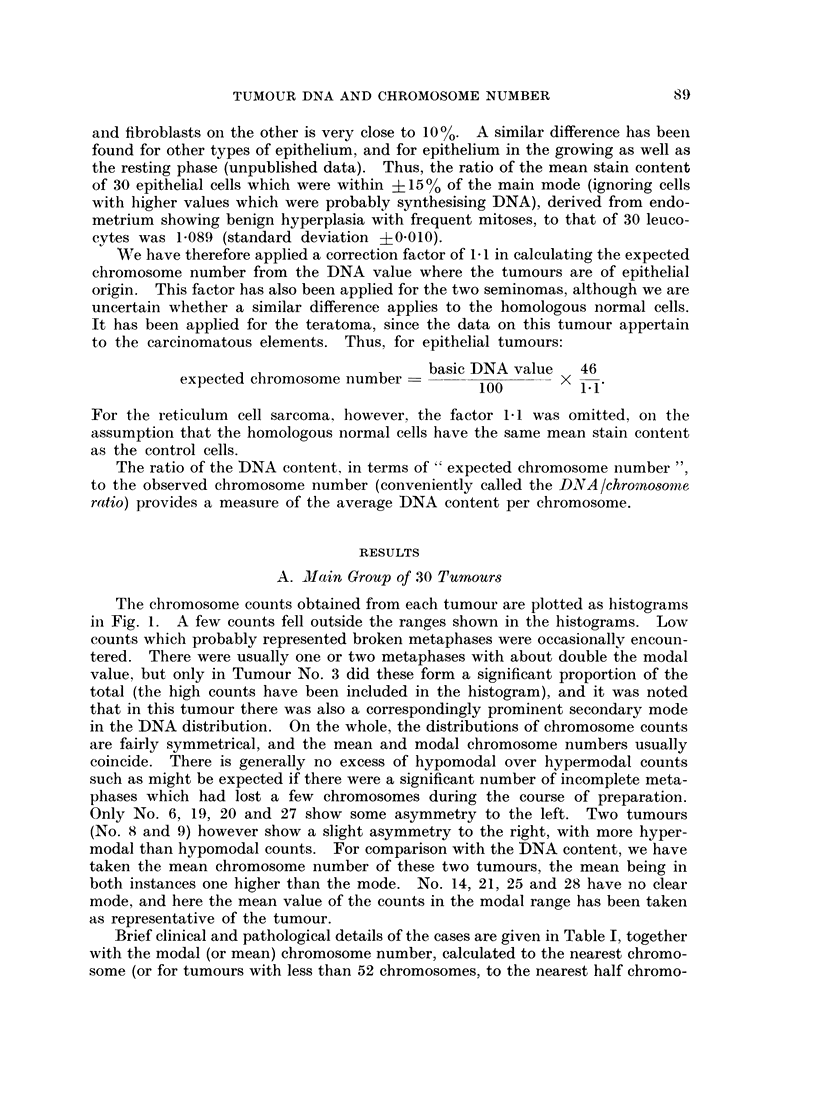

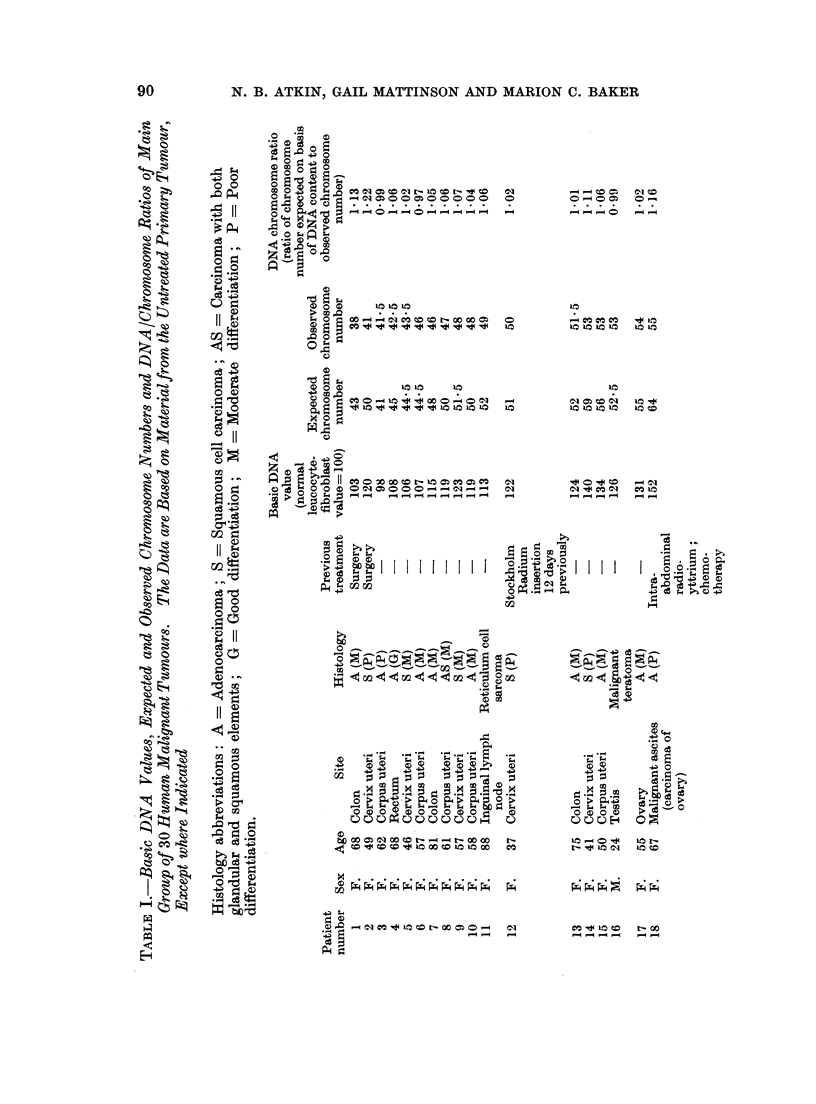

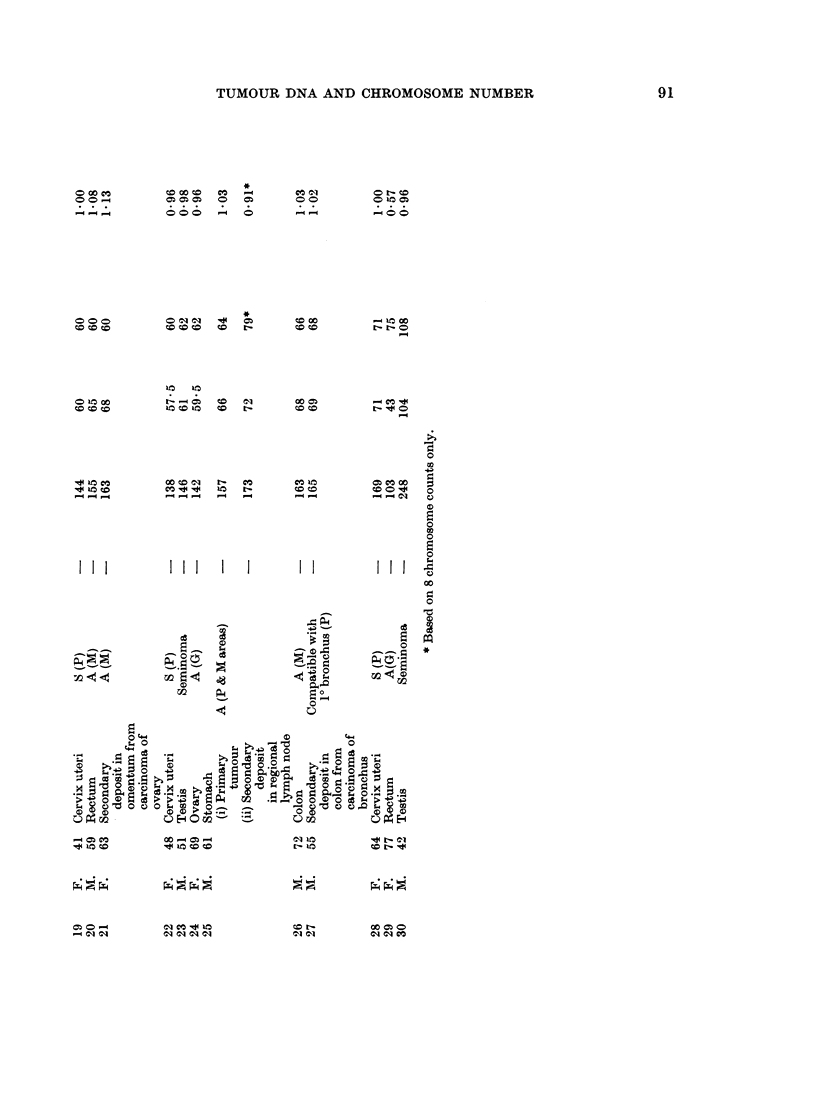

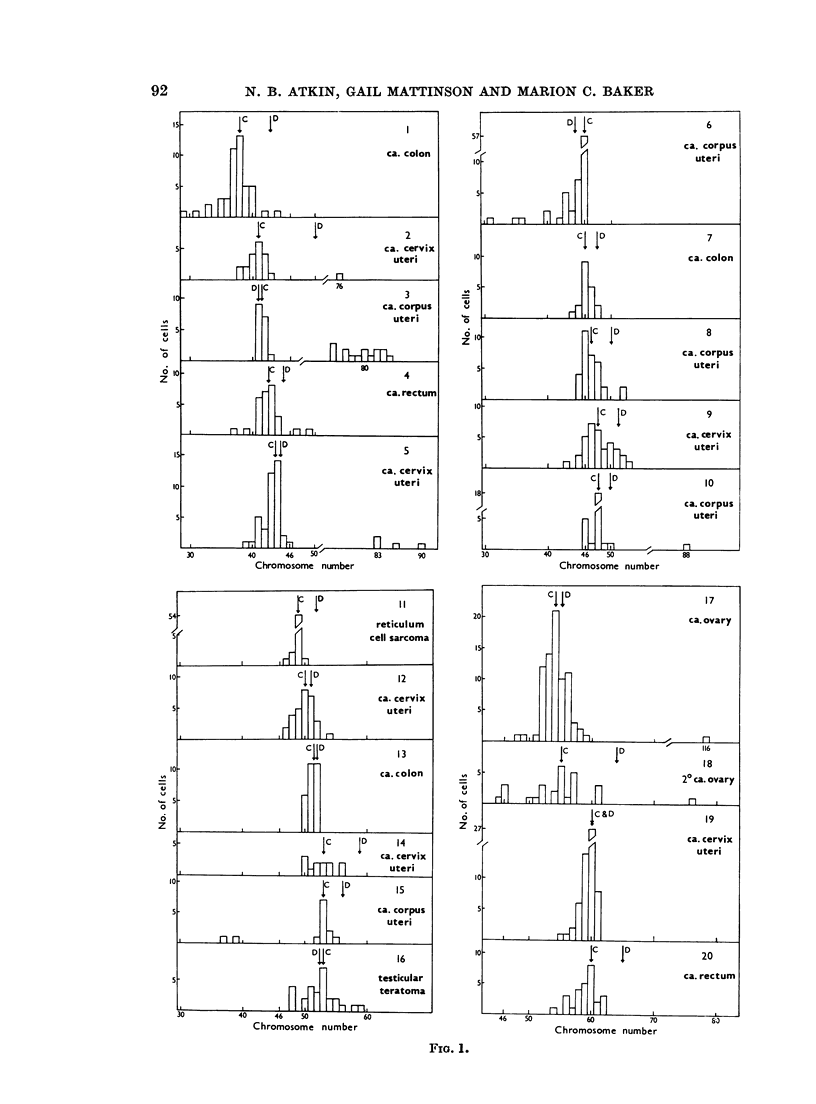

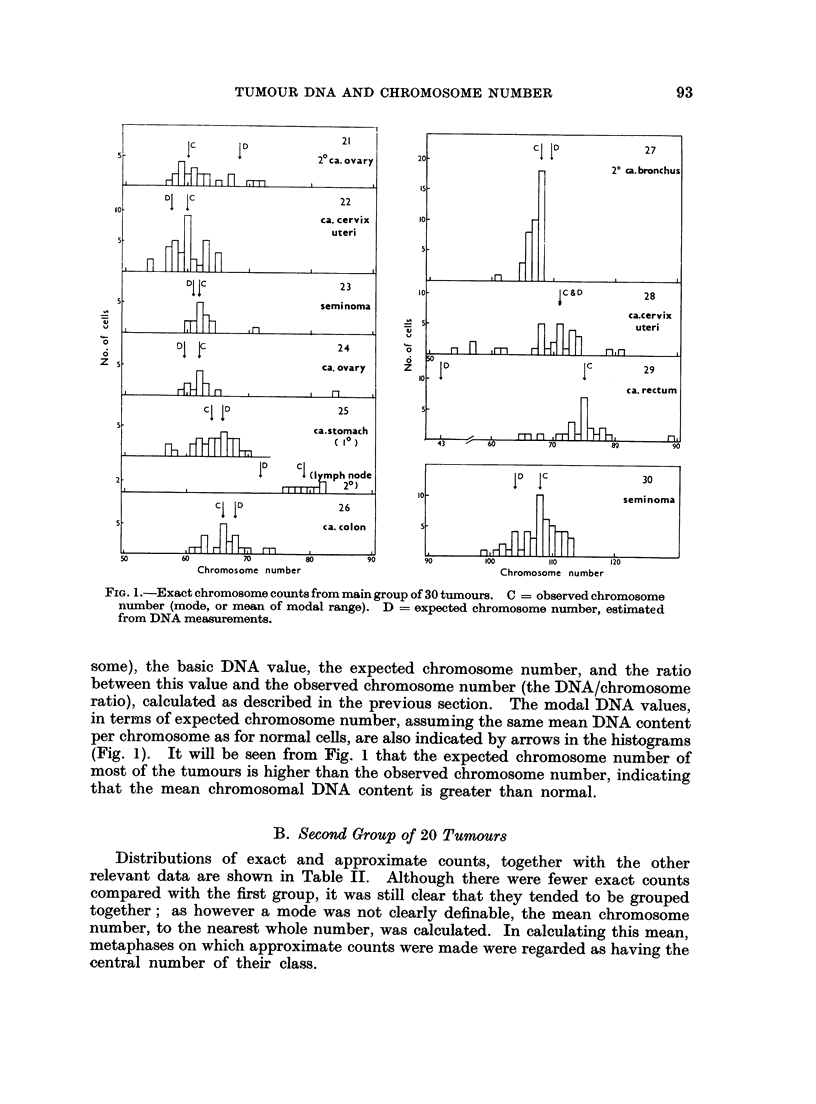

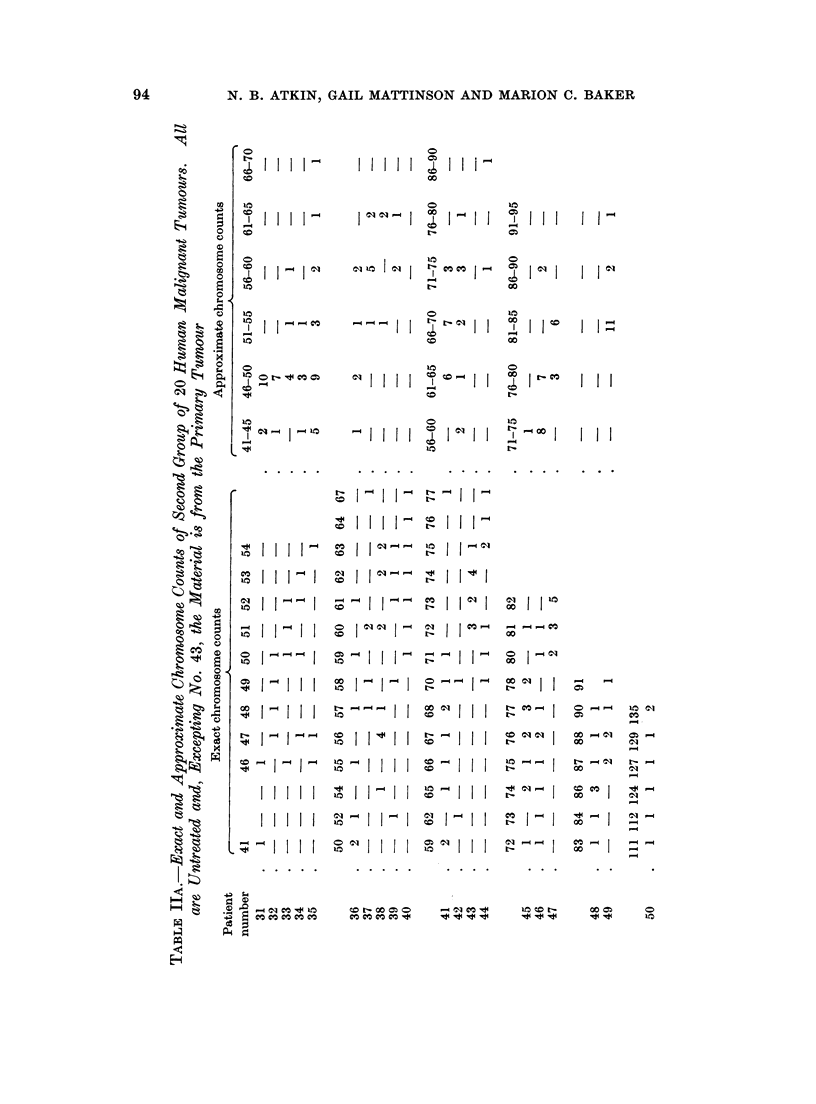

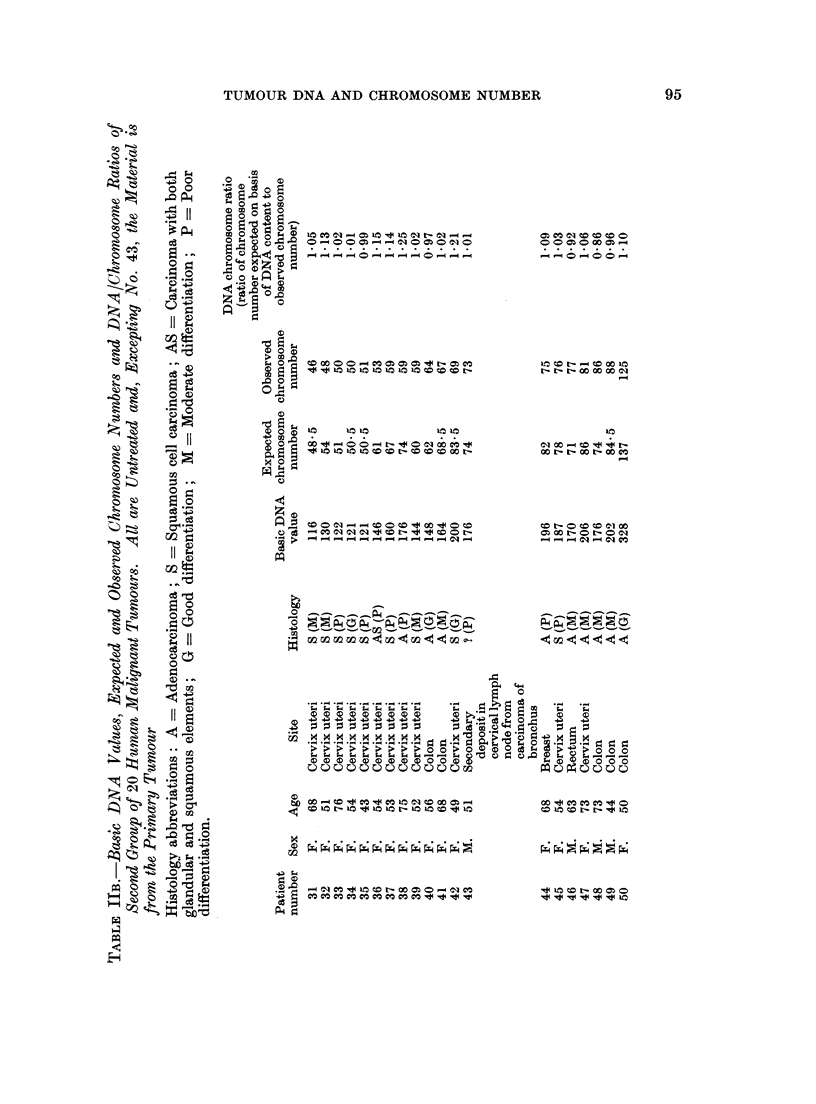

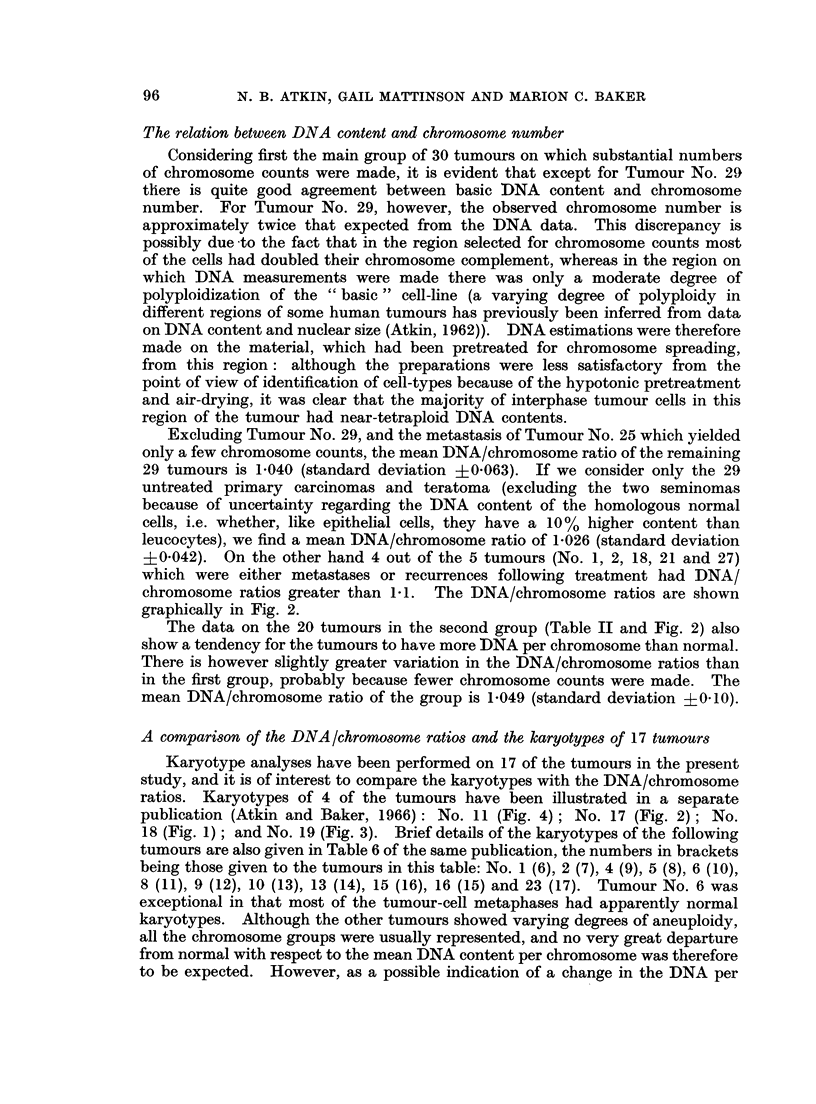

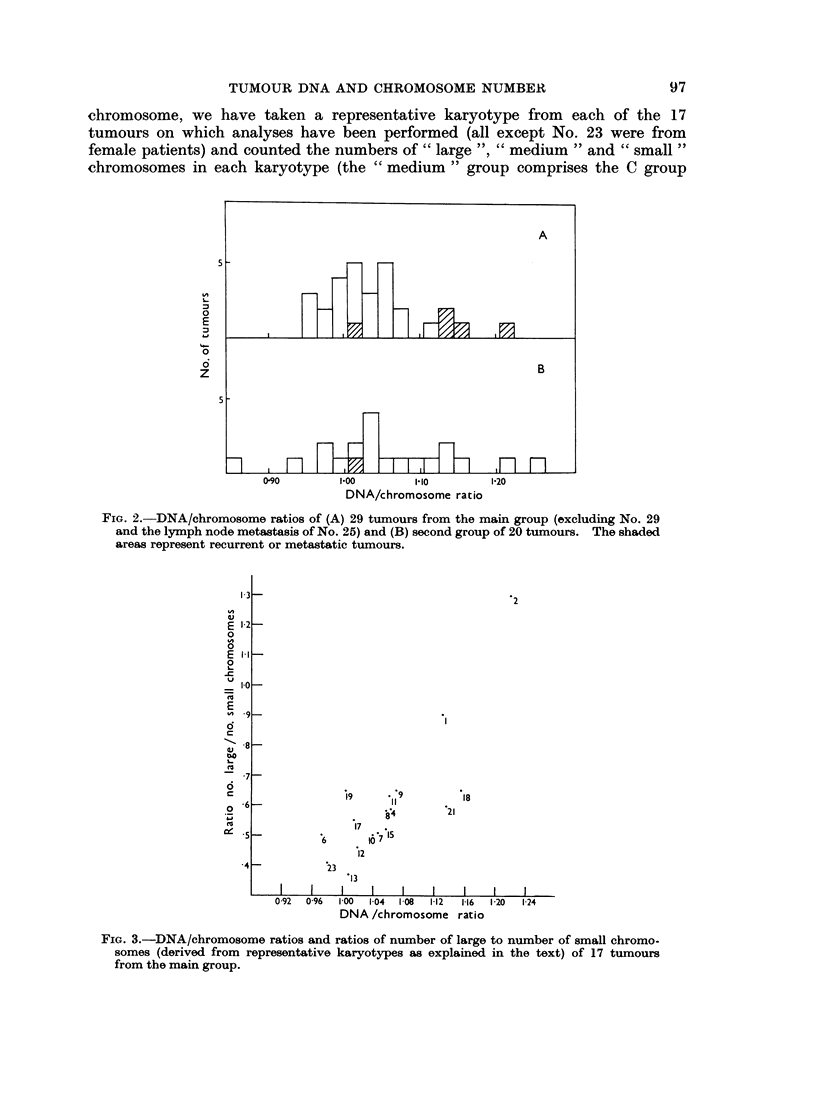

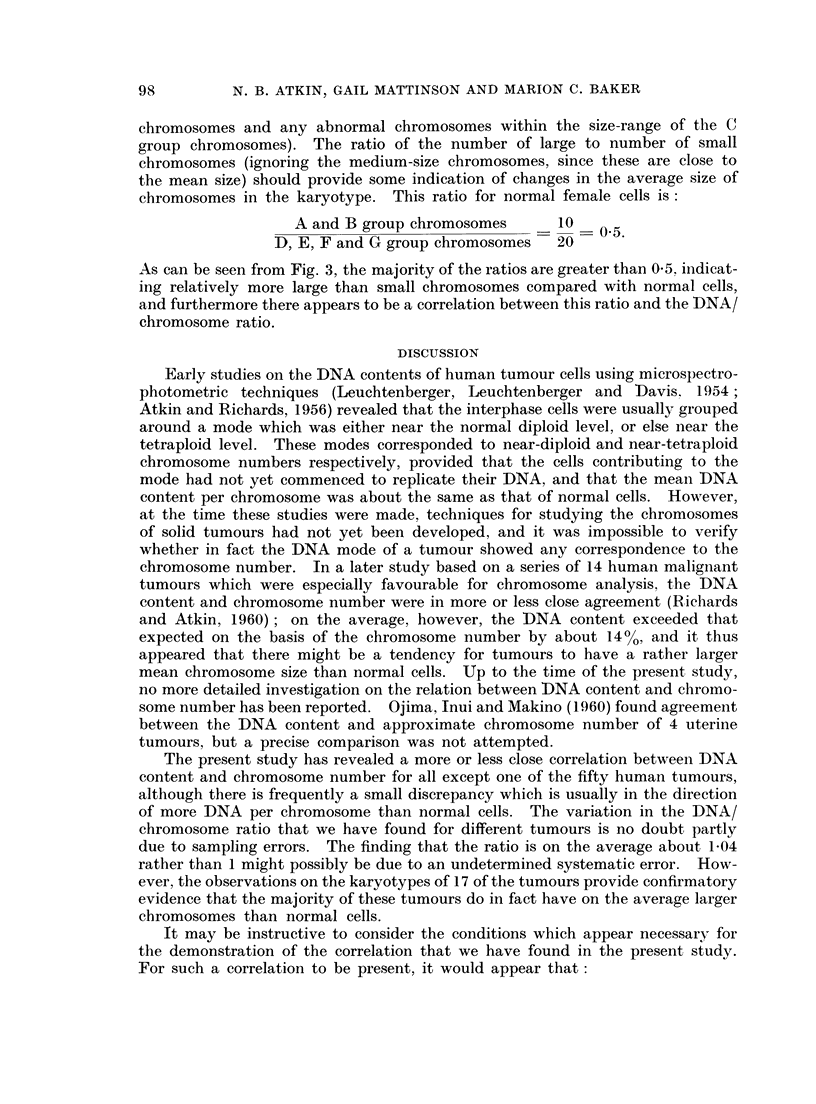

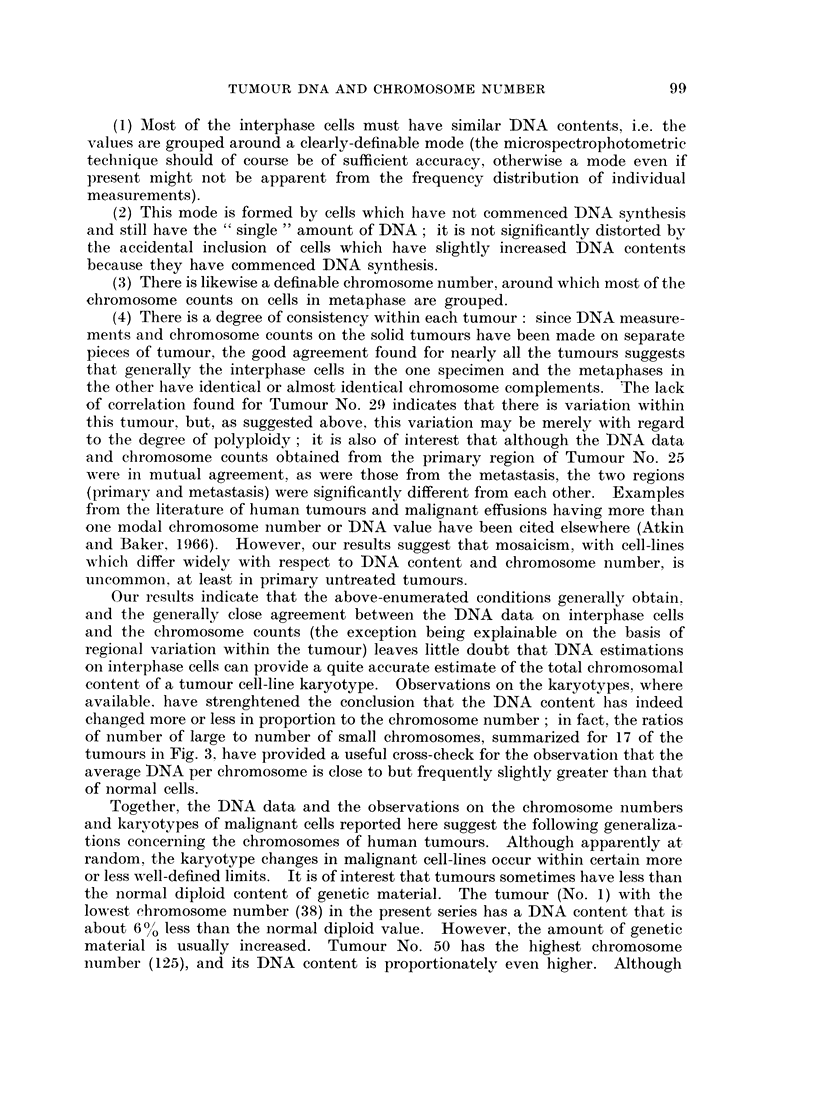

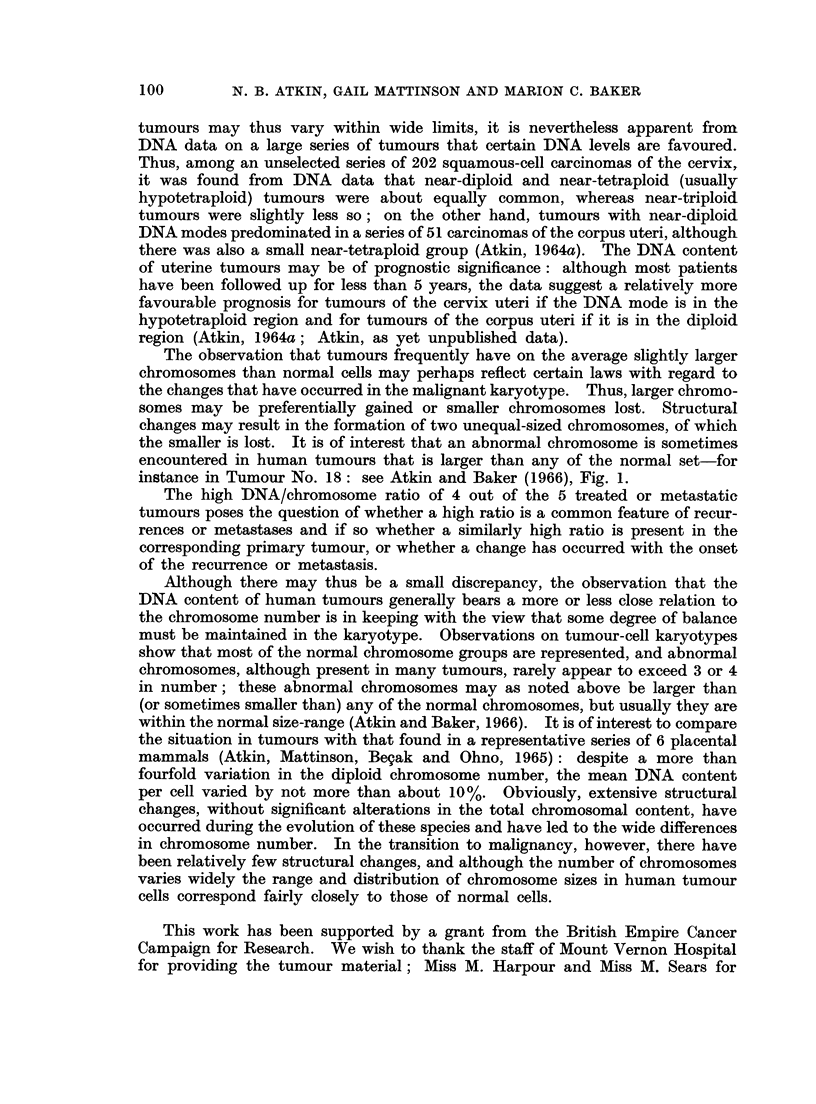

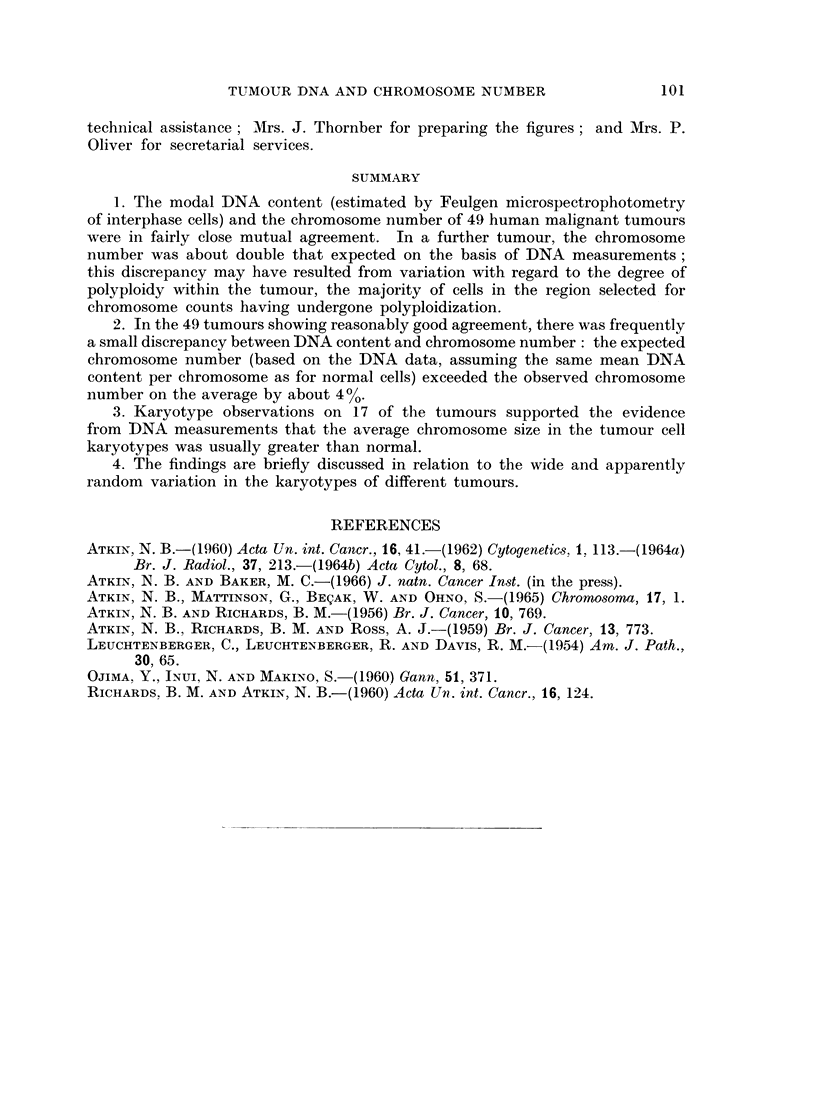

